# *In silico *analysis of chimeric *espA*, *eae *and *tir *fragments of *Escherichia coli *O157:H7 for oral immunogenic applications

**DOI:** 10.1186/1742-4682-6-28

**Published:** 2009-12-08

**Authors:** Jafar Amani, S Latif Mousavi, Sima Rafati, Ali H Salmanian

**Affiliations:** 1 National Institute of Genetic Engineering and Biotechnology (NIGEB), Shahrak-e- Pajoohesh, 15th Km, Tehran -Karaj Highway, Tehran, IR Iran; 2 Baqiyatallah University of Medical Science, Department of Biotechnology, Tehran, IR Iran; 3 Department of Biology, Faculty of Basic Sciences, Shahed University, Tehran, IR Iran; 4 Molecular Immunology and Vaccine Research Laboratory, Department of Immunology, Pasteur Institute of Iran, Tehran, IR Iran

## Abstract

**Background:**

*In silico *techniques are highly suited for both the discovery of new and development of existing vaccines. *Enterohemorrhagic Escherichia coli *O157:H7 (EHEC) exhibits a pattern of localized adherence to host cells, with the formation of microcolonies, and induces a specific histopathological lesion (attaching/effacing). The genes encoding the products responsible for this phenotype are clustered on a 35-kb pathogenicity island. Among these proteins, Intimin, Tir, and EspA, which are expressed by attaching-effacing genes, are responsible for the attachment to epithelial cell that leads to lesions.

**Results:**

We designed synthetic genes encoding the carboxy-terminal fragment of Intimin, the middle region of Tir and the carboxy-terminal part of EspA. These multi genes were synthesized with codon optimization for a plant host and were fused together by the application of four repeats of five hydrophobic amino acids as linkers. The structure of the synthetic construct gene, its mRNA and deduced protein and their stabilities were analyzed by bioinformatic software. Furthermore, the immunogenicity of this multimeric recombinant protein consisting of three different domains was predicted.

**Conclusion:**

a structural model for a chimeric gene from LEE antigenic determinants of EHEC is presented. It may define accessibility, solubility and immunogenecity.

## Background

*Enterohemorrhagic Escherichia coli *O157:H7 (EHEC) is an important human pathogen [1], causing diarrhea and in some cases hemolytic-uremic syndrome (HUS), leading to kidney failure and even death [2]. EHEC produces several virulence factors, enabling it to colonize the large bowel and cause disease [3].

Cattle are most frequently identified as the primary source of bacteria, so reduction in *E. coli *O157:H7 prevalence in cattle by vaccination represents an attractive strategy for reducing the incidence of human disease [4]. An experimental vaccine was recently shown to significantly reduce shedding of the organism under natural exposure conditions [5].

These pathogenic bacteria contain a chromosomal island known as the Locus of Enterocyte Effacement (LEE, 35KD), containing genes critical for forming the attachment and effacement (A/E) lesion. This locus can be divided into three functional regions: the first one encoding a type III secretion system; the second containing the genes *eae *and *tir; *and the third consisting of *espD*, *espB*, and *espA *[6,7].

Intimin, a key colonization factor for EHEC O157:H7 acts as an outer membrane adhesion protein which is encoded by the gene *eae*. This protein mediates bacterial attachment through its C-terminal region to enterocytes by binding to Tir (Translocated Intimin Receptor) [8,9].

Tir, a 78-kDa protein, is secreted from EHEC and is efficiently delivered into the host cell [10,11].

The type III secretion system is involved in the secretion of different proteins including EspA, EspB, EspD, and Tir. EspA forms a filamentous structure on the bacterial surface as a bridge to the host cell surface. It delivers EspB, EspD, and Tir directly into the host cell. EspB is delivered primarily into the host cell membrane where it becomes an integral membrane protein and, along with EspD, forms a pore structure through which other bacterial effectors, such as Tir, enter the host cell [6,12]. Additionally, studies on rabbit models indicate that pedestal formation is mediated by the same proteins (Intimin, EspA, EspB, EspD and Tir), and translocated Tir can bind to intimin via amino acids 258 to 361 [3,13].

The Tir-Intimin interaction causes attachment of EHEC to the intestinal cell surface and triggers actin cytoskeletal rearrangements, resulting in pedestal formation. Recent evidence shows that active immunization of mice with recombinant Intimin from *Citrobacter rodentium *as a mouse model pathogen can prevent colonization of bacteria in the digestive tracts of animals [14].

These determinants are potent mucosal immunogens and induce humoral and mucosal responses (IgA instead of IgG) following oral administration [15,16]. Among different systems for oral administration, transgenic plants are becoming more attractive because of their low cost, easy scale-up of production, natural storage organs (tubers and seeds), and established practices for efficient harvesting, storing, and processing [17,18]. Moreover, a number of proteins such as recombinant antibodies and recombinant subunit vaccines have been expressed successfully in transgenic plants [19].

In this study we designed a new structural model containing three putative antigenic determinants of EspA, Intimin and Tir, fused together by hydrophobic linkers. Addition of the regulatory sequences Kozak and ER-retention signal at the 5' and 3' ends respectively, and codon optimization of this chimeric gene for expression in plants, were used to improve the efficiency of transcription and translation [20-22]. Finally, a novel *in silico *approach was used to analyze the structure of the designed chimeric protein.

## Results

### Design and construction of chimeric gene

The 282 amino acids from the carboxy terminus of Intimin have been reported to be involved in binding to its receptor Tir [23,24]. The region of Tir involved in the interaction with intimin has also been mapped (residues 258 to 361, designated Tir 103) [25]. For the third fragment, a truncated form of *espA *(lacking 36 amino acids from the N-terminal of the protein, designated EspA 120) was selected. This part of EspA120 is exposed on the bacterial surface [6].

Upon sequence comparison by ClustalW, the C-terminals of intimin (282 amino acids) and EspA (120 amino acids) and the middle part of Tir (103 amino acids) showed high degree of conservation among different strains of *E. coli *O157:H7 (Data not shown).

**Figure 1:**
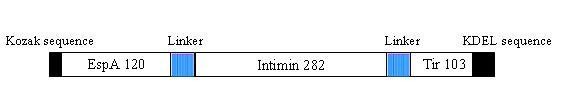
**Schematic model which shows the construction of EspA 120, Intimin 282 and Tir 103, bound together by the linkers for expression in plants; these fragments were selected on the basis of the common sequence found in different strains of *E. coli *O157 H7**.

These three parts were selected for designing a synthetic construct. In order to separate the different domains, linkers consisting of EAAAK repeats and expected to form a monomeric hydrophobic α-helix were designed. It has been shown that the salt bridge Glu^-^-Lys^+ ^between repeated Ala can stabilize helix formation [26]. Four repeated EAAAK sequences were introduced between different domains for more flexibility and efficient separation. The Kozak sequence [27] was added before the start codon in order to ensure high and accurate expression of mRNA in a eukaryotic host. For efficient accumulation of the recombinant protein in Endoplasmic Reticulum (ER), the sequence KDEL was added at the end of the synthetic construct. Arrangements of fragment junctions and linker sites are shown in Figure 1.

### Bioinformatic analysis of the wild type and optimized synthetic gene

**Figure 2:**
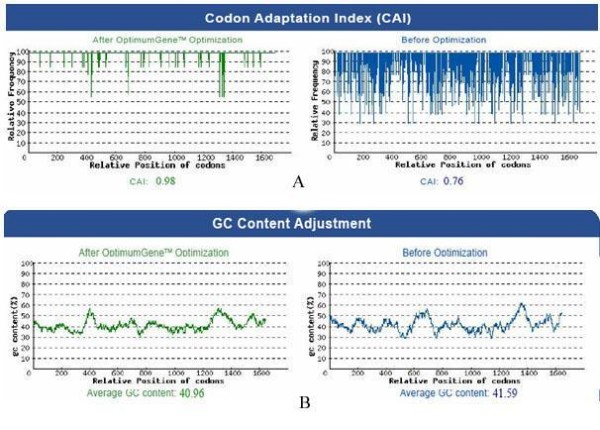
**A: Codon usage analysis of wild type and optimized gene for expression in plants**. The value of 100 is set for the codon with the highest usage frequency for a given amino acid in the desired expression into plants. This procedure allows us to compare the adaptiveness of different codons relative to each other (*relative adaptiveness*). Plots represent the relative adaptiveness of a given codon at the indicated codon position. B: GC analysis of wild type and optimized chimeric gene. Plots represent the average GC content, before and after optimization.

A synthetic sequence encoding the chimeric gene was designed using plant codon bias. To optimize the synthetic gene, negatively *cis *acting motifs and repeated sequences were avoided. Both the wild type and the synthetic chimera were analyzed for their codon bias (Figure 2A) and GC content (Figure 2B),

The overall GC content was reduced from 41.59 to 40.96%, which should increase the overall stability of mRNA from the synthetic gene. Moreover, there was no sequence stretch within the gene showing an average GC content below 40%.

The optimized gene showed a codon bias for plants and contained no rarely used codon. This is also reflected by the codon adaptation index (CAI), which is a measurement of the relative adaptiveness of the codon usage of a gene compared with the codon usage of highly expressed genes. The chimeric gene showed a CAI of 0.98, compared to that of the wild type gene, which was only 0.76 [28].

Within the synthetic construct, the splice sites, polyadenylation signal, instability elements, and all the *cis*-acting sites that may have a negative influence on the expression rate were removed (Table 1). Furthermore, the necessary restriction enzyme sites (*Xb*aI and *Sac*I) were introduced at the ends of the sequence for cloning purpose.

**Table 1:** Analysis of cis-acting elements

Splice site	Original	Optimized
GGTAAG	2	0

GGTGAT	4	0

GTAAAA	1	0

GTAAGT	2	0

GTACGT	0	0

**Poly A**		

AATAAA	1	0

AATGAA	0	0

AATGGA	0	0

TATAAA	2	0

AATAAT	3	0

AAAAAAA	1	0

**Poly T**		

TTTTTT	1	0

**Destabilizing element**		

ATTTA	3	0

### mRNA structure prediction

A genetic algorithm-based RNA secondary structure prediction was combined with comparative sequence analysis to determine the potential folding of the chimeric gene. The 5' terminus of the gene was folded in the way typical of all bacterial gene structures. The minimum free energy for secondary structures formed by RNA molecules was also predicted. All 34 structural elements obtained in this analysis revealed folding of the RNA construct. The data showed the mRNA was stable enough for efficient translation in the new host (Data not shown) [29].

### Protein secondary structure prediction

**Figure 3:**
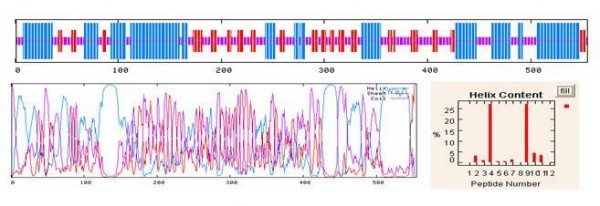
**Analysis of chimeric EspA-Intimin-Tir protein secondary structure**.

The secondary structure of the chimeric protein was predicted by online software. Three prediction methods were compared for evaluating the structure of this protein. The results showed that helix structures lie in the regions of aa 129 to 148 and aa 431 to 450, which are related to the hydrophobic amino acids inserted between different domains (Figure 3) [30,31].

### Tertiary structural prediction for the chimeric protein

**Figure 4:**
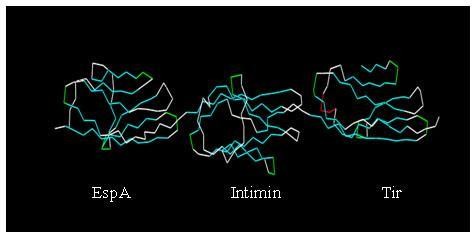
***Ab initio *and comparative modeling was used to predict the tertiary structure of the chimeric protein, EspA-Intimin-Tir**. The result was viewed by Rasmol software.

Comparative and *ab initio *modeling of the synthetic sequence was exploited to produce 3D models of the chimeric protein. Two hundred thirty three-dimensional models were generated for this chimeric protein. The models were uploaded to the server to draw the tertiary structural illustrations with Swiss-PdbViewer and Rasmol software in order to determine the final structure of the protein. Furthermore, SCRATCH servers http://www.igb.uci.edu/ developed by California University were used for protein structure prediction by PSI-BLAST and neural networks. There were two α-helices and several β-turns, which were consistent with the results of secondary structure analyses. The results of tertiary structure prediction showed the formation of three separate domains of the chimeric protein (Figure 4) [32,33].

### Evaluation of model stability

**Figure 5:**
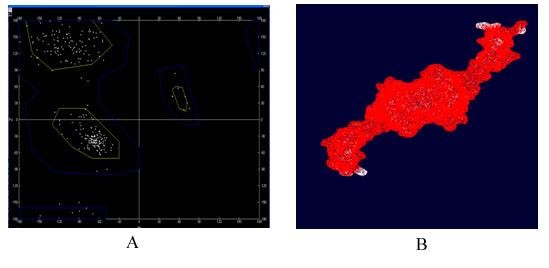
**(A) Evaluation of model stability based on a Ramachandran plot and (B) energy minimization**.

The profile of energy minimization was calculated by spdbv (Swiss-PdbViewer) (-1391.230 Kcal/mol) indicating that the recombinant protein had acceptable stability compared to that of original structure of each domain. Additionally, the data generated by a Ramachandran plot confirmed the structural stability of the protein (Figure 5).

### Solvent accessibility prediction

The solvent accessibility distributions were characterized using the major hydrophobic and polarity properties of residual patterns. These patterns showed that the mean residue accessible surface area (ASA) gave a high solvent accessibility value, approximately fifty percent (Data not shown) [34].

### Prediction of B-cell epitopes

Different factors such as hydrophilicity, plasticity, exterior accessibility, antigenicity and secondary structure were used to predict the chimeric protein epitopes. The epitopes located on the surface of the protein could interact easily with antibodies, and they were generally flexible. Bcepred software was used to determine the continuous B cell epitope based on single characters including hydrophilicity, antigenicity, flexibility, accessibility, polarity and exposed surface (Table 2). As shown in Table 2, linkers between different domains (aa 129 to 148 and aa 431 to 450) contained no epitope sites [35-37]. Furthermore, the conformational epitopes for B cells were predicted by the Discotope server (Table 3) [38].

**Table 2:** Epitopes predicted in chimeric protein by different parameters based on Bcepred software

Prediction parameters	Epitope positions*
Hydrophilicity	1-14, 25-38, 47-55, 108-115, 128-144, 160-166, 202-219, 222-230, 232-242, 262-268, 283-291, 301-309, 319-329, 392-404, 448-475, 482-490, 512-526, 528-547, 430-446.

Flexibility	4-10, 25-35, 43-51, 104-113, 199-214, 217-226, 279-287, 307-314, 316-325, 389-403, 447-453, 480-485, 539-545.

Accessibility	2-18, 27-42, 45-55, 81-87, 95-101, 113-120, 128-144, 147-155, 157-166,169-177, 179-191, 201-217, 250-259, 276-282, 289-298, 319-331, 340-349, 374-384, 391-401, 430-463, 467-493, 510-551.

Exposed surface	28-42, 251-259, 340-346, 392-398, 450-457, 472-479, 482-490, 520-527, 530-550.

Polarity	32-39, 128-144, 157-164, 249-259, 430-446, 473-480, 510-526, 533-552.

Antigenic propensity	38-44, 112-119, 174-180, 312-319, 352-360, 363-370, 413-419, 498-508.

* Number shows position of amino acids.

**Table 3:** One hundred and eighteen discontinuous B-Cell epitopes of chimeric protein predicted by the Discotope server

Start & End position	Start & End position	Start & End position	Start & End position	Start & End position	Start & End position	Start & End position	Start & End position	Start & End position
49-6	174-19	202-19	252-12	312-10	351-10	377-11	389-8	412-14

50-13	175-21	204-16	253-17	324-14	359-11	378-10	390-5	425-5

51-13	176-12	229-8	254-4	325-13	361-13	379-12	391-8	426-5

52-17	177-11	231-6	255-8	326-9	364-14	380-13	392-11	427-7

54-15	178-13	232-7	256-8	327-13	367-12	381-11	402-12	433-11

55-16	179-21	242-12	257-9	328-12	368-8	382-10	403-7	439-11

69-9	189-10	243-12	258-9	329-12	369-9	383-8	404-9	

70-8	193-12	244-16	259-9	330-12	370-9	384-5	405-10	

72-9	194-12	245-13	260-11	331-14	371-12	385-6	406-8	

89-15	195-17	246-12	276-10	345-7	372-10	386-7	407-8	

90-14	196-14	247-11	277-12	346-7	373-11	387-6	408-11	

136-8	197-13	249-18	281-12	347-7	374-12	388-10	409-10	

137-7	200-19	250-17	286-12	349-7	375-12	389-8	410-15	

138-9	201-21	251-13	287-8	350-11	376-12	388-10	411-14	

## Discussion

Many bacterial pathogens infect or invade their hosts via mucosal surfaces. This process is initiated by the attachment of the bacteria to the cell membrane via specific receptors. Enterohemorrhagic *E. coli *is a good model and has been well studied in this context. In this bacterium, the antigens Intimin, EspA, and Tir are required for attachment to the intestinal mucosa [39]. If the function of these receptors was impaired, the bacterium could not attach to the host cell surface and the disease would be suppressed. This impairment is related to the production of immunoglobulin class A (IgA), which is the dominant antibody on the mucosal surface [2].

Therefore, mucosal immunization especially via the oral route is an attractive strategy for inducing protective immunity against mucosal pathogens [40]. Several vehicles (Polymers, Alginate, Polyphosphazenes and other biodegradable polymers, Immunostimulating complexes (ISCOM), Liposomes) [41] have been used for delivering antigen to the target tissue. The capacity of plants for producing vaccines which could induce mucosal immunity is a great advantage. Plant cells act as a natural microencapsulation system to protect the vaccine antigens from being degraded in the upper digestive tract before they can reach the gut-associated lymphoid tissue (GALT) [18]. Studies on B subunit labile toxin (LTB) suggest that plant-based oral vaccines can significantly boost mucosal immune responses that have been primed by parenteralinjection [42].

One the most important problems in transgenic plants is low level production of recombinant immunogenic protein. To solve this problem, different strategies such as strong promoter, organelle targeting and organelle transformation have been used [17]. Furthermore, synthetic genes with plant codon optimization have been used to mimic highly expressed plant genes. The effective applications of synthetic genes in plants have been proven by other researchers [16].

Two types of vaccines are available against *E. coli *O157:H7: one is a genetically engineered vaccine tested on a small group of adult volunteers. It appears safe and stimulates the production of antibodies against the potentially fatal pathogen [43]. The other is *Econiche *(made from an extract of lysed bacteria containing type III secretion proteins) for vaccination of healthy cattle as an aid in reducing shedding of *Escherichia coli *O157: H [44]. Both of these vaccines are high risk and are insufficiently safe and for this reason we attempted to design multi component antigens which can create protection and prevent colonization. This construct should contain essential antigenic factors of *E. coli *O157:H7 that are exposed completely.

On the basis of knowledge of molecular modeling and immuno-informatics, a novel approach was employed to identify a set of peptides that could be used as a vaccine either in natural or in synthetic form. This approach has been extended to the entire proteomes of other microorganisms such as T-cell epitopes of secretory proteins of *Mycobacterium tuberculosis *[45,46], Tertiary Structure of *Mycobacterium leprae *Hsp65 Protein [47], T-cell antigen of *Chlamydia *[48], tandem repeat antigens from *Leishmania donovani *[49], and Envelope Glycoprotein of *Japanese Encephalitis Virus *(JEV) [50] to identify new sets of potentially antigenic proteins.

Here we designed new constructs of EHEC antigens including EspA, Intimin and Tir that contained essential determinants for bacterial attachment and effacement. Theoretically, the DNA fragment consisted of these three putative antigens and could be synthesized as a unique construct optimally suited for expression in a plant system. Several factors which can affect the expression of foreign genes in plant systems such as messenger RNA instability [51], premature polyadenylation [52], abnormal splicing [53], and improper codon usage have been reported [54]. In order to increase the mRNA stability, DNA motifs that might contribute to mRNA instability in plants, such as the ATTTA sequence and the potential polyadenylation signal sequence AATAAA, were eliminated from the synthetic gene (for detail see Table 1). The synthetic DNA fragment which encoded the mature chimeric gene was constructed based on the codon usage of highly expressed nuclear-encoded genes of tobacco *(Nicotiana tobaccum L.) *as a model, and canola (*Brassica napus L*.) as the final target plant [55].

The efficiency of heterologous protein production can be diminished by biased codon usage. Approaches normally used to overcome this problem include targeted mutagenesis to remove rare codons or the addition of rare codon tRNAs in specific cell lines. Recently, improvements in the technology have enabled synthetic genes to be produced cost-effectively, making this a feasible alternative [56]. In addition, as each step in the process of gene expression, from the transcription of DNA into mRNA to the folding and posttranslational modification of proteins, is regulated by complex cellular mechanisms, a relationship is expected to exist between mRNA expression levels and protein solubility in the cell. By formulating a relation between the mRNA expression level and the recombinant protein, production can be reasonably predicted [57].

In eukaryotic mRNA, the consensus sequence surrounding the start codon (Kozak seq. 5'GCC ACCATGGC) can increase the correctness and efficiency of translation up to 10 fold. In the synthetic construct, the 5'GCCACC sequence was added before the ATG codon. The second codon following the initial methionine was Ala, encoded by the codon GCT, and the necessary GC was provided; therefore there was no need to replace the other nucleotides or amino acids [27]. Codons that are rarely used in plants, such as XCG and XUA (X denotes U, C, A, or G), were avoided in the construction of the synthetic gene (Figure 2B). It has been reported that rare codons in mRNA tend to form higher-order secondary structures, which might require additional time for ribosomal movement through the critical region [58].

An ideally biased gene would show a codon adaptation index (CAI) of 1.0. Even though no natural plant gene reaches this theoretical value, this index was increased from 76% in the wild type chimeric sequence to 98% in this synthetic gene. Furthermore, the G/C ratio and distribution were balanced from 41.59 to 40.96 percent with no significant changes, and this has been reported to be associated with low mRNA stability and expression in higher plants [55]. The nucleotide that encodes the ER retention signal (KDEL) which helps to accumulate the recombinant protein inside the endoplasmic reticulum was fused in-frame at the 3^' ^end of the chimeric gene [15,16]. Finally, the required restriction enzyme sites (*Xba*I and *Sac*I) were introduced at the ends of the synthetic gene for future cloning into plant expression vectors.

Graphical depiction of the predicted minimum free energy for the synthetic gene showed that the average energy minimization was near - 400 Kcal/mol.

Comparison of the synthetic gene with the original one revealed no major difference between these two molecules and their structures were compatible with each other.

In the protein structure prediction, the chimeric protein formed three domains that were separated by two main α-helix moieties which could help the protein to form a final structure. These α-helix structures are related to the designation of special amino acid sequences, residues 129-148 and 431-450, which are inserted between domains. With these results we could speculate that these parts could support the stable structure of a protein which contained three domains.

B-cell epitopes for the chimeric protein could be predicted on the basis of the structural prediction and solvent accessibility. Hopp and Woods in the 1980s developed a method for predicting B-cell epitopes with hydrophilicity parameters. Since then, several distinct methods such as Hydrophilicity method, Accessibility method, Antigenicity method, Flexibility method and secondary structure analysis have been developed [36,39]. Applying just one of these methods is not enough for obtaining results good enough to predict the B-cell epitope. In this study, we combined all the data obtained by these analyses and predicted the B-cell epitopes.

The integrated results showed that the most likely B-cell epitopes of this chimeric protein, as shown in Table 2, were located in three distinct parts, selected as the EspA, Intimin, and Tir domains.

For eliciting an immune response against *E. coli *O157:H7, studies have shown that production of the carboxy terminal part of Intimin in a transgenic plant cell line and its application via the oral route is more effective than injection [16]. In this study, we designed a multi domain antigen which was selected on the basis of three immunogenic parts of attaching/effacing loci from *E. coli *O157:H7, which were then optimized upon plant codon preference for analyzing mucosal and systematic immunity.

## Conclusion

Bioinformatics tools for predicting epitopes are now a standard methodology. In silico epitope mapping, combined with in vitro and in vivo verification, accelerates the discovery process by approximately 10-20-fold. Development of sophisticated bioinformatics tools will provide a platform for more in-depth analysis of immunological data and facilitate the construction of new hypotheses to explain the complex immune system function [59].

In this study, we have combined several techniques and profiles to improve the state-of-the-art prediction of 3D structure and relative solvent accessibility. Building a homology model for this chimeric protein has been used to understand the antigenic sites and structural conformation domains which were used to predict continuous and discontinuous epitopes. Also, for the antibody-antigen interaction, it is important to know how much area of surface is exposed; accordingly we defined the exposed areas and surface accessibility.

Considering the multi colonization factor of this bacterium, multi antigenic parts should be used for repressing this pathogen. For this reason, more research should focus on designing multi antigenic proteins from *E. coli*O157:H7. This study and a few others [60,61] indicate that epitope construction and prediction will be useful not only in vaccine development but also in the prospective engineering and re-engineering of protein therapeutics, reducing the risk of undesired immunogenecity and improving the likelihood of success in clinical use.

Finally, the conclusions drawn for *E. coli *O157:H7 proteins could be combined with expression profiling to identify genes whose expression changes under shifting environmental conditions [62].

In conclusion, we believe that all of these findings will intensify efforts to develop a vaccine candidate against *E. coli *O157:H7.

## Methods

### Sequence analysis

Related sequences for *espA *(40 sequences), *eae *(32 sequences) and *tir *(50 sequences) were obtained from Genbank (accession no. not shown). Multiple sequence alignments were performed using ClustalW software (EBI, UK) http://www.ebi.ac.uk/Tools/clustalw2/ in order to identify a fragment common to all the sequences.

### Construct design

An antigenic sequence was constructed by fusing the C-terminal of *espA*, C-terminal of *eae *and middle fragment of *tir *using hydrophobic amino acid linkers (accession no. GQ205376).

The *in silico *gene analysis and multi parameter gene optimization of the synthetic chimera gene was performed using Stand-alone softwares such as Leto (Entelechon, Germany), DNA 2.0 http://www.dnatwopointo.com, DNAsis MAX (Hitachi Software), and online data bases and softwares such as the codon database http://www.kazusa.or.jp/codon, Gene bank codon data base and Swissprot reverse translation online tool http://www.bioinformatics.org/sms2/rev_trans.html. The desired properties were verified by Gen-Script (NJ, USA). The multimeric gene was synthesized by ShineGene Molecular Biotech, Inc (Shanghai, China).

### Bioinformatic analysis of chimeric recombinant protein

The messenger RNA secondary structure of the chimeric gene was analyzed by the program mfold http://www.bioinfo.rpi.edu/applications/mfold. Recombinant protein Secondary-structure predictions were performed by the neural-network-based algorithm program (PHD), and for 3D structure, online *ab initio *software was used http://www.igb.uci.edu/[63]. 3D structural stability of the synthetic protein was further analyzed by Swiss-PdbViewer for energy minimization [64]. Solvent accessibility of different residues was evaluated by DSSP and other online programs (VADAR) http://redpoll.pharmacy.ualberta.ca/vadar/. The predictive value of the hyper glycosylation code which may act in plants is well established based on online software http://www.cbs.dtu.dk/services/[65].

### Prediction of B-cell epitopes

The amino acid sequence was analyzed using three web-based B-cell epitope prediction algorithms; Bcepred http://www.imtech.res.in/raghava/bcepred/, Continuous B cell epitopes prediction methods based on physico-chemical properties on a non-redundant dataset, and the Discotope http://www.cbs.dtu.dk/services/DiscoTope/ Server for predicting discontinuous B cell epitopes from three-dimensional protein structures. Briefly, chimeric proteins were analyzed first for continuous B-cell epitopes using Bcepred and then using the Discotope server to predict discontinuous B cell epitopes. Finally, we used the VaxiJen server to predict the immunogenecity of the whole antigen and its subunit vaccine [48,66,67].

## Competing interests

The authors declare that they have no competing interests.

## Authors' contributions

All four authors (JA, SLM, SR, AHS) contributed equally to this manuscript. All authors read and approved the final manuscript.
